# A pilot study on the impact of maternal diet and preconception body mass index on breast milk macronutrient composition

**DOI:** 10.1186/2049-3258-72-S1-P3

**Published:** 2014-06-06

**Authors:** Goele Jans, Rivka Turcksin, Bart Van der Schueren, Christophe Matthys, Roland Devlieger

**Affiliations:** 1 Department of Development and Regeneration, KU Leuven, Leuven, 3000, Belgium; 2 Faculty of Medicine, Nursing and Midwifery, KU Leuven, Leuven, 3000, Belgium; 3 Department of Clinical and Experimental Medicine, KU Leuven, Leuven, 3000, Belgium; 4 Department of Endocrinology, UZ Leuven, Leuven, 3000, Belgium; 5 Department of Obstetrics and Gynecology, UZ Leuven, Leuven, 3000, Belgium

## Background

Breastfeeding is the number one recommendation to serve newborns and young children with all nutrients they need for a healthy growth and development. The aim of this pilot study was to investigate the impact of maternal diet and preconception Body Mass Index (BMI) on the macronutrient composition of breast milk.

## Materials and methods

A cross-sectional study was conducted at the maternity units of the University Hospital of Leuven, Belgium. Postpartum women, who delivered a term (≥37 weeks of gestation), did not smoke, were normoglycaemic and who were not on predefined medication, were invited to participate. Participants completed a 24 dietary recall and donated a breast milk sample of 1.5-2cl at day 4 post-delivery. The samples were collected during the first feeding in the morning by use of a vacuum pump. Each sample was analyzed for macronutrients (carbohydrates, protein, fats) and energy with the MIRIS® Human Milk Analyzer. The pre-pregnancy weight and length were assessed from the online medical patient file to calculate the pre-pregnancy BMI. Further baseline characteristics included maternal age, ethnicity and parity. Pregnancy outcomes included delivery mode, the use of combined spinal epidural, gestational age, birth weight and gender of the baby.

## Results

Analyses have been performed on samples from 33 postpartum women. There were no differences in baseline characteristics. A positive correlation was found between maternal BMI and carbohydrate concentration in breast milk (r=0.3778; p=0.030). A significant difference in human milk carbohydrate concentration was seen when dividing into groups, indicating a higher concentration in obese women (BMI ≥30 kg/m²) compared to normal weight women (BMI 18.5-24.9 kg/m²) (7.3±0.9 g/100ml versus 6.6±0.6 g/100ml; p=0.017). A linear regression showed that every increase of one unit of BMI presented an increase of 0.045g in mean carbohydrate concentration. No correlation was found between the maternal diet and the concentration of macronutrients and energy in breast milk.

## Conclusions

Breast milk composition partially depends from maternal BMI, with only a higher carbohydrate concentration in the obese women. The maternal diet seems to have no impact on the macronutrient milk composition. This is not consistent with results of other studies, reporting an impact of maternal fat consumption on the fat composition of breast milk. The small study group does not allow drawing strong conclusions. Analyses with a larger study group are needed.

